# Implementing Preventive Chemotherapy through an Integrated National Neglected Tropical Disease Control Program in Mali

**DOI:** 10.1371/journal.pntd.0001574

**Published:** 2012-03-20

**Authors:** Massitan Dembélé, Sanoussi Bamani, Robert Dembélé, Mamadou O. Traoré, Seydou Goita, Mamadou Namory Traoré, Abdoul Karim Sidibe, Letitia Sam, Marjon Tuinsma, Emily Toubali, Chad MacArthur, Shawn K. Baker, Yaobi Zhang

**Affiliations:** 1 Programme National d'Elimination de Filariose Lymphatique du Mali, Bamako, Mali; 2 Programme National de Lutte contre la Cécité du Mali, Bamako, Mali; 3 Programme National de Lutte contre les Schistosomiases et les Géohelminthiases du Mali, Bamako, Mali; 4 Programme National de Lutte contre l'Onchocercose du Mali, Bamako, Mali; 5 Helen Keller International, Bamako, Mali; 6 Direction Nationale de la Santé, Ministère de la Santé, Bamako, Mali; 7 Helen Keller International Headquarters, New York, New York, United States; 8 Helen Keller International, Regional Office for Africa, Dakar, Senegal; Centers for Disease Control and Prevention, United States of America

## Abstract

**Background:**

Mali is endemic for all five targeted major neglected tropical diseases (NTDs). As one of the five ‘fast-track’ countries supported with the United States Agency for International Development (USAID) funds, Mali started to integrate the activities of existing disease-specific national control programs on these diseases in 2007. The ultimate objectives are to eliminate lymphatic filariasis, onchocerciasis and trachoma as public health problems and to reduce morbidity caused by schistosomiasis and soil-transmitted helminthiasis through regular treatment to eligible populations, and the specific objectives were to achieve 80% program coverage and 100% geographical coverage yearly. The paper reports on the implementation of the integrated mass drug administration and the lessons learned.

**Methodology/Principal Findings:**

The integrated control program was led by the Ministry of Health and coordinated by the national NTD Control Program. The drug packages were designed according to the disease endemicity in each district and delivered through various platforms to eligible populations involving the primary health care system. Treatment data were recorded and reported by the community drug distributors. After a pilot implementation of integrated drug delivery in three regions in 2007, the treatment for all five targeted NTDs was steadily scaled up to 100% geographical coverage by 2009, and program coverage has since been maintained at a high level: over 85% for lymphatic filariasis, over 90% for onchocerciasis and soil-transmitted helminthiasis, around 90% in school-age children for schistosomiasis, and 76–97% for trachoma. Around 10 million people have received one or more drug packages each year since 2009. No severe cases of adverse effects were reported.

**Conclusions/Significance:**

Mali has scaled up the drug treatment to national coverage through integrated drug delivery involving the primary health care system. The successes and lessons learned in Mali can be valuable assets to other countries starting up their own integrated national NTD control programs.

## Author Summary

Neglected tropical diseases (NTDs) are a group of chronic infections that affect the poorest group of the populations in the world. There are currently five major NTDs targeted through mass drug treatment in the affected communities. The drug delivery can be integrated to deliver different drug packages as these NTDs often overlap in distribution. Mali is endemic with all five major NTDs. The integrated national NTD control program was implemented through the primary health care system using the community health center workers and the community drug distributors aiming at long-term sustainability. After a pilot start in three regions in 2007 without prior examples to follow on integrated mass drug administration, treatment for the five targeted NTDs was gradually scaled up and reached all endemic districts by 2009, and annual drug coverage in the targeted population has since been maintained at a high level for each of the five NTDs. Around 10 million people received one or more drug treatments each year since 2009. The country is on the way to meet the national objectives of elimination or control of these diseases. The successes and lessons learned in Mali are valuable assets to other countries looking to start similar programs.

## Introduction

Neglected tropical diseases (NTDs) are a group of diseases that affect the most vulnerable and the poorest group of the populations in the world [1], [2]. The World Health Organization (WHO) recommends five public health strategies for the prevention and control of the NTDs: preventive chemotherapy (PCT); intensified case management; vector control; provision of safe water, sanitation and hygiene; and veterinary public health [1]. The major NTDs currently being targeted through PCT include lymphatic filariasis (LF), onchocerciasis, schistosomiasis, soil-transmitted helminthiasis (STH, including ascariasis, hookworm infection and trichuriasis) and trachoma. These five major NTDs cause high disease burden with severe disfigurement, disability and blindness, blighting the lives of more than one billion people worldwide and threatening the health of millions more [1]. The drugs needed for these five NTDs are robust, safe, low-cost and available by donation from the pharmaceutical companies or by purchasing at relatively low costs [3]. They can be delivered to the target populations either alone or in combination to prevent morbidity caused by these NTDs, or in some cases, to eliminate the diseases [4], [5].

Mali is landlocked in West Africa with a population of 15.5 million. It is divided into eight administrative regions (Kayes, Koulikoro, Sikasso, Segou, Mopti, Tombouctou, Gao and Kidal) and Bamako. The northern part of the country extends deep into the Sahara desert and the southern region features the Niger and Senegal rivers, where the majority of the country population inhabits. The country's economy centers on agriculture and fishing. Mali is one of the poorest countries in the world and ranked 160 out of 169 countries according to the Human Development Report 2010 [6]. It is endemic with all five major NTDs [7]–[11]. Control of the NTDs before 2007 was through four independent vertical national programs under the Ministry of Health (MoH): the National Onchocerciasis Control Program (PNLO), the National Lymphatic Filariasis Elimination Program (PNEFL), the National Schistosomiasis and Soil-Transmitted Helminths Control Program (PNLS) and the National Blindness Prevention Program (PNLC).

Onchocerciasis was originally prevalent in five regions in the country, including Kayes, Koulikoro, Sikasso, Segou and Mopti, and the PNLO was established in 1986 to address the public health implications of the disease. The eastern part of the endemic regions (Koulikoro rive droite, Sikasso, Ségou and Mopti) was included in the original program area of the Onchocerciasis Control Program (OCP). In 2002 onchocerciasis was declared eliminated as a public health program in large parts of these areas with only epidemiological and entomological surveillance continuing to monitor the prevalence and microfilarial load in the population and to also monitor the infectivity of the vector *Simulium damnosum*. The western part of the endemic regions (Kayes and Koulikoro rive gauche) was included in the western extension of OCP in 1987 with ivermectin (IVM, donated by Merck & Co.) administration and later with Community Directed Treatment with Ivermectin (CDTI) with support from the African Program for Onchocerciasis Control (APOC) and using the community drug distributors (CDDs). The disease is currently endemic in 17 districts (Sikasso, one of the original 16 districts, was split into two separate districts in 2010) in three regions in Kayes, Koulikoro and Sikasso.

LF, caused by *Wuchereria bancrofti*, is endemic throughout Mali [10], [12] with the entire population being at risk of disease. The PNEFL was established in 2004 and subsequently a national mapping survey was carried out using Immunochromatographic Test cards confirming LF endemicity across Mali (Dembélé, unpublished data). The MDA for LF started in 2005 in four of the five onchocerciasis districts in Sikasso using CDTI plus albendazole (ALB, donated by GlaxoSmithKline), with support from the Government of Mali.

Both urogenital (caused by *Schistosoma haematobium*) and intestinal (caused by *S. mansoni*) forms of schistosomiasis are present in the country [13]. Two national surveys were conducted with the first in 1984–1989 and the second in 2004–2006 [7], [14], [15]. The results confirmed presence of schistosomiasis throughout the country with geographically varying degrees of prevalence. The later survey in 2004–2006 showed a prevalence of 38.3% (ranging 0.0–99.0%) for *S. haematobium* and 6.7% (ranging 0.0–94.9%) for *S. mansoni* [15]. Schistosomiasis control in Mali was initiated in the Bandiagara district, Mopti as a component of a dam-building project in 1978 and became a national program (PNLS) in 1982 [13], [15]. The initial control program with praziquantel (PZQ) distribution was implemented by the MoH in collaboration with WHO and with support from the German Technical Co-operation [13], but the MDA ceased later due to lack of further funding. In 2005, the MDA resumed with support from the Schistosomiasis Control Initiative (SCI) with PZQ procured from certified generic manufacturers, targeting school-age children and at-risk adults with PCT through school-based and community-based drug delivery in all endemic regions and Bamako (school-age children only) [16], [17].

STH is a public health problem throughout Mali. The national survey in 2004–2006 (together with schistosomiasis) in school children from 7–14 years of age showed that the whole country is endemic for STH, with prevalence of up to 34.3% with hookworm infection (in Yorosso, Sikasso) (R Dembélé, unpublished data). STH control consists of several drug delivery platforms in Mali. The National Intensified Nutrition Weeks (SIAN, French acronym) deliver vitamin A and ALB twice a year to children of 12–59 months and to women immediately post-partum. In 2004, the PNLS was expanded to include STH, and ALB was delivered at the same time through school-based and community-based drug delivery to those receiving PZQ treatments for schistosomiasis during 2005–2007 with the support from the Schistosomiasis Control Initiative. The population above 5 years also benefits from annual treatment with ALB and IVM from the LF elimination program.

Trachoma as a blinding disease is found in all districts of the eight regions of the country (except Bamako). A national survey in all regions except Bamako was conducted in 1996–1997 [11]. The prevalence of active trachoma, follicular (TF) or intense (TI), was estimated to be 34.9% among children under 10 years of age, and the prevalence of trichiasis among women over 14 years of age was 2.5%, and 1% had central corneal opacity [11]. The PNLC initiated a trachoma control program in 1998 following the national survey adopting the WHO recommended SAFE (Surgery, Antibiotics, Facial washing and Environmental improvement) strategy [18], [19], benefiting from the Zithromax (ZTM) donation program by Pfizer Inc. Significant progress had been achieved in trachoma control since the start of the national program [19]–[21].

As one of the five ‘fast-track’ countries supported by the United States Agency for International Development (USAID) NTD Control Program managed by RTI International [22], Mali launched the integrated national NTD Control Program (NTDCP) in 2007 with technical assistance initially from International Trachoma Initiative (ITI, 2007) and then from Helen Keller International (HKI) from 2008 onward. The overall objectives of the Mali's NTD control program are to eliminate LF, onchocerciasis and trachoma as public health problems and to reduce morbidity caused by schistosomiasis and STH through regular mass drug administration (MDA), with specific objectives of achieving 80% program coverage and 100% geographical coverage yearly within the five-year program plan. This current paper serves as a report on the progress made by the integrated national NTDCP in Mali, drawing from objectives achieved, documented experiences and pertinent lessons learned of the program from 2007 to 2011, and focusing on only aspects of integrated MDA activities.

## Methods

### The national integrated NTD Control Program

The existing disease-specific vertical national programs achieved various degrees of coverage throughout the country and mapping of distribution of each NTD was almost complete before integration. These disease-specific national control programs already achieved significant success before 2007 as described in the introduction. Integration of control activities on certain diseases already occurred, e.g. onchocerciasis and LF, and schistosomiasis and STH, on co-delivery of drugs. Building on these successes, in 2007 Mali began to further integrate the existing disease-specific control programs to increase efficiency and program coverage for each target disease. The USAID funds support all the integrated PCT-related activities and procurement of PZQ. Although the integrated NTD control program include other non-MDA components, this paper focuses on the implementation of MDA component only.

### Leadership

The NTDCP is led by the MoH through the National Directorate of Health. The National Steering Committee of the program was established and is chaired by the National Director of Health and its members include members of the Technical Coordinating Committee (TCC, described below), the Head of Planning, Training and Health Information Unit, the Head of Public Health and Safety Division, the Head of the Nutrition Division, the Dean of the Faculty of Medicine, Pharmacy and Odonto-Stomatology (FMPOS), and the representatives of non-governmental developmental organization (NGDO) partners. The Steering Committee meets twice a year to discuss the progress of the program and issues arising.

A National Strategic Plan for integrated control of NTDs (2007–2011) was developed in 2007 as the blueprint to direct the control activities. A new five-year national strategic plan (2012–2016) is being updated and finalized.

### Program coordination

Under the National Directorate of Health, Division of Disease Control and Prevention (DPLM) is responsible for coordinating the activities of control and elimination of priority diseases in Mali. The existing four disease-specific national control programs are under the remit of DPLM, which provides an ideal framework for coordination of integrated NTD control activities. The dedicated NTD coordinator at HKI works closely with the four National Coordinators of the disease-specific control programs to facilitate the integrated activities.

**Figure 1:**
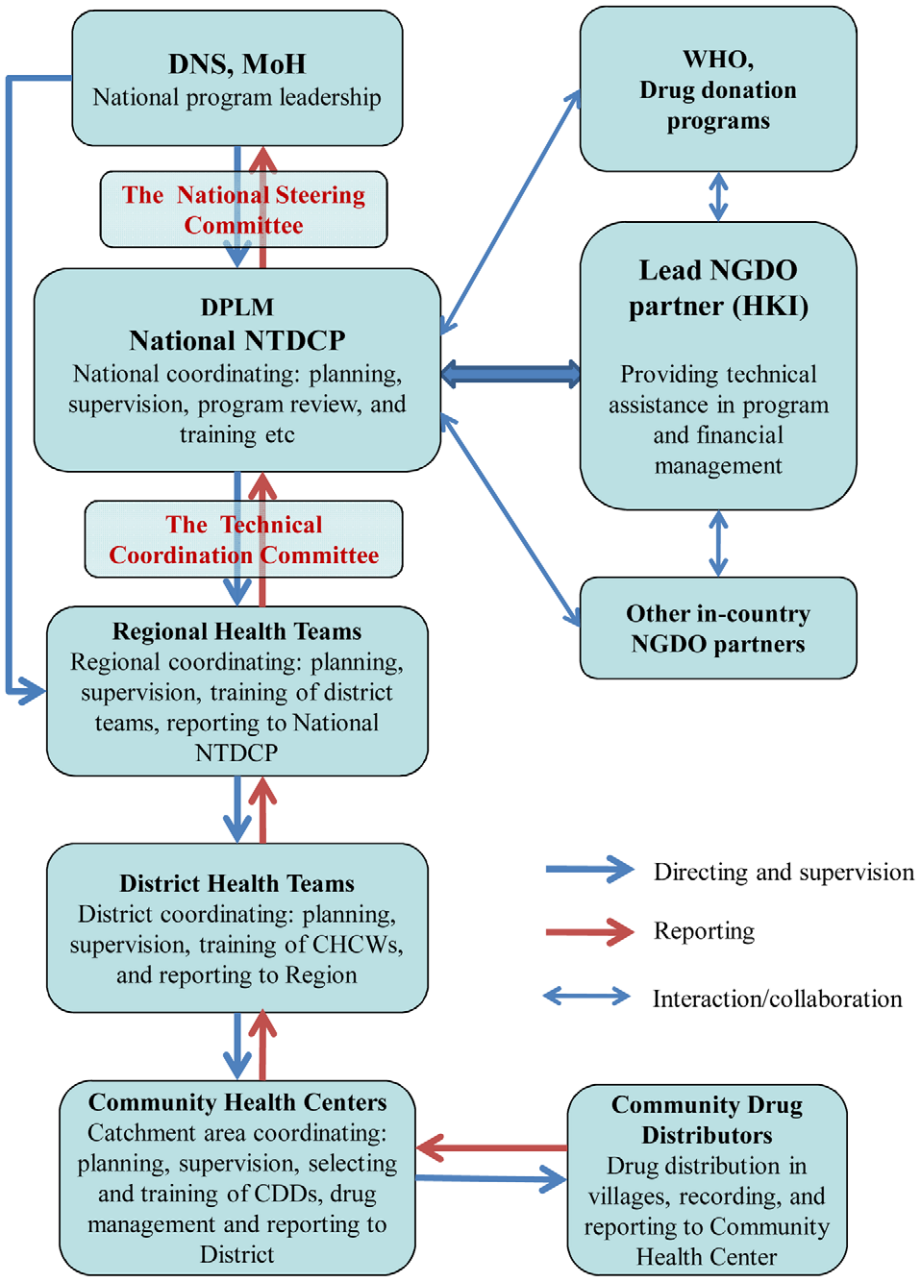
The NTD control program coordination structure in Mali.

Under the DPLM, the TCC was established and is chaired by the Chief of DPLM, comprising four National Coordinators of the disease-specific control programs, the Head of Nutrition Division, the representative from the National Public Health Research Institute, the representative from the National Center of Information, Education and Communication for Health (CNIECS), and the representative of the grantee NGDO (initially ITI and currently HKI). This committee meets every quarter. The program review and planning workshop was conducted annually to review the progress and to plan for the coming year, attended by the TCC members, the Regional Health Directors, and the regional NTD focal persons. The Regional Health Teams in turn planned the MDA activities for each district with the District Health Teams. In Mali, community health centers play a very important role in providing primary health care at local level. Within each district, there are a number of community health centers, each responsible for a number of villages. For long-term sustainability and local capacity building, the NTD control activities were integrated into the primary health care system at local level. Community health center workers (CHCWs) play an important role in the program as their routine health care activities. These CHCWs provided training and supervision of CDDs, and were responsible for drug allocation, treatment data collation in their catchment area, and data reporting to the district health officers. Figure 1 shows the structure of the program.

### Implementation strategies

**Figure 2:**
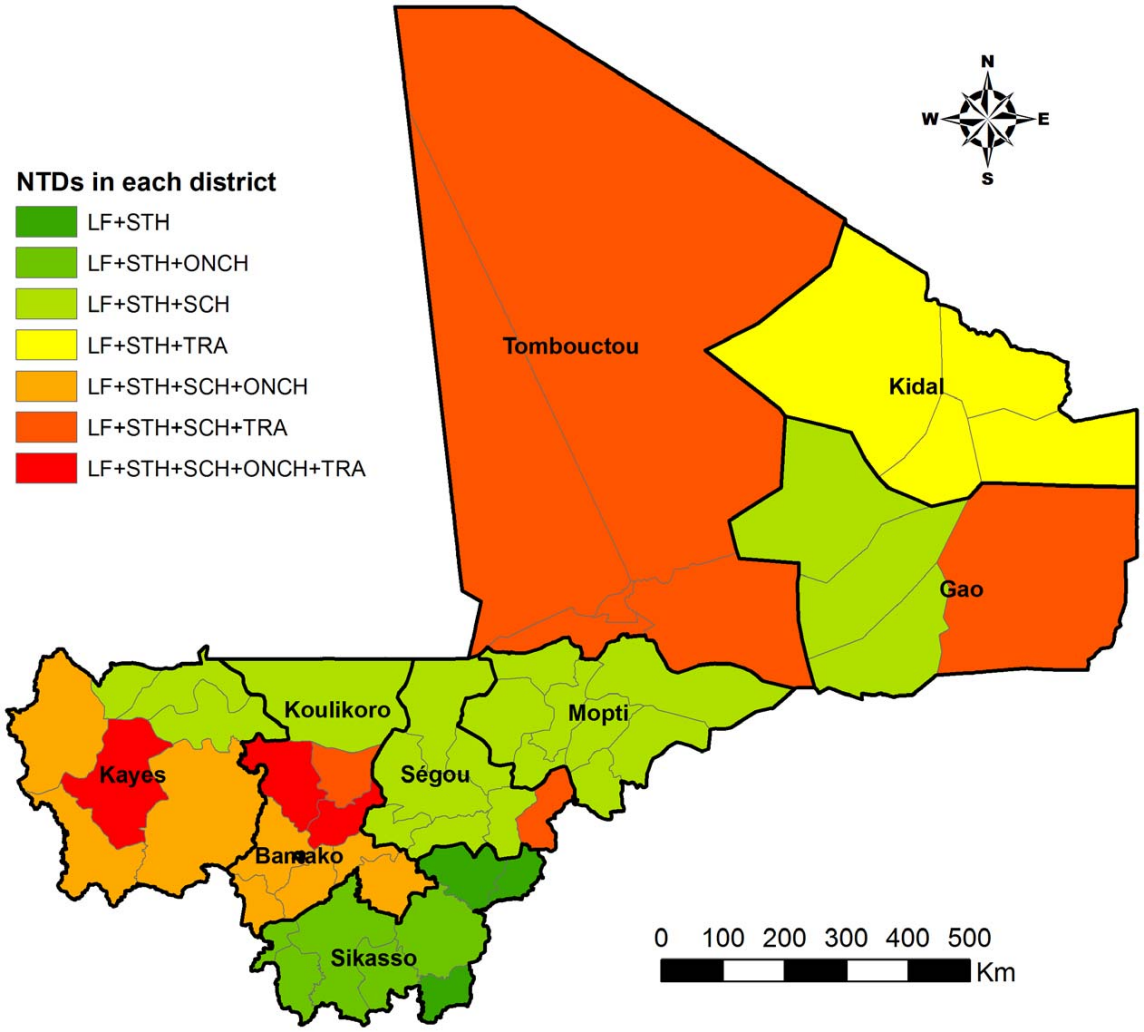
Endemic situation shown as number of major target NTDs in each district in Mali. LF: lymphatic filariasis; ONCH: onchocerciasis; SCH: schistosomiasis; STH: soil-transmitted helminthiasis; TRA: trachoma. In Kidal region, the endemicity level of schistosomiasis in each district is not yet clear and further mapping is planned.

To integrate the PCT activities of each existing control program, a situation analysis was conducted to map out the overlaps of the disease distribution in each district using the existing disease distribution data. Figure 2 shows the overlapping distributions of the five targeted NTDs in each districts of the country. The PCT strategy for each disease in each district was decided according to the known prevalence of the disease in the district and the WHO PCT guidelines [4].

**Figure 3:**
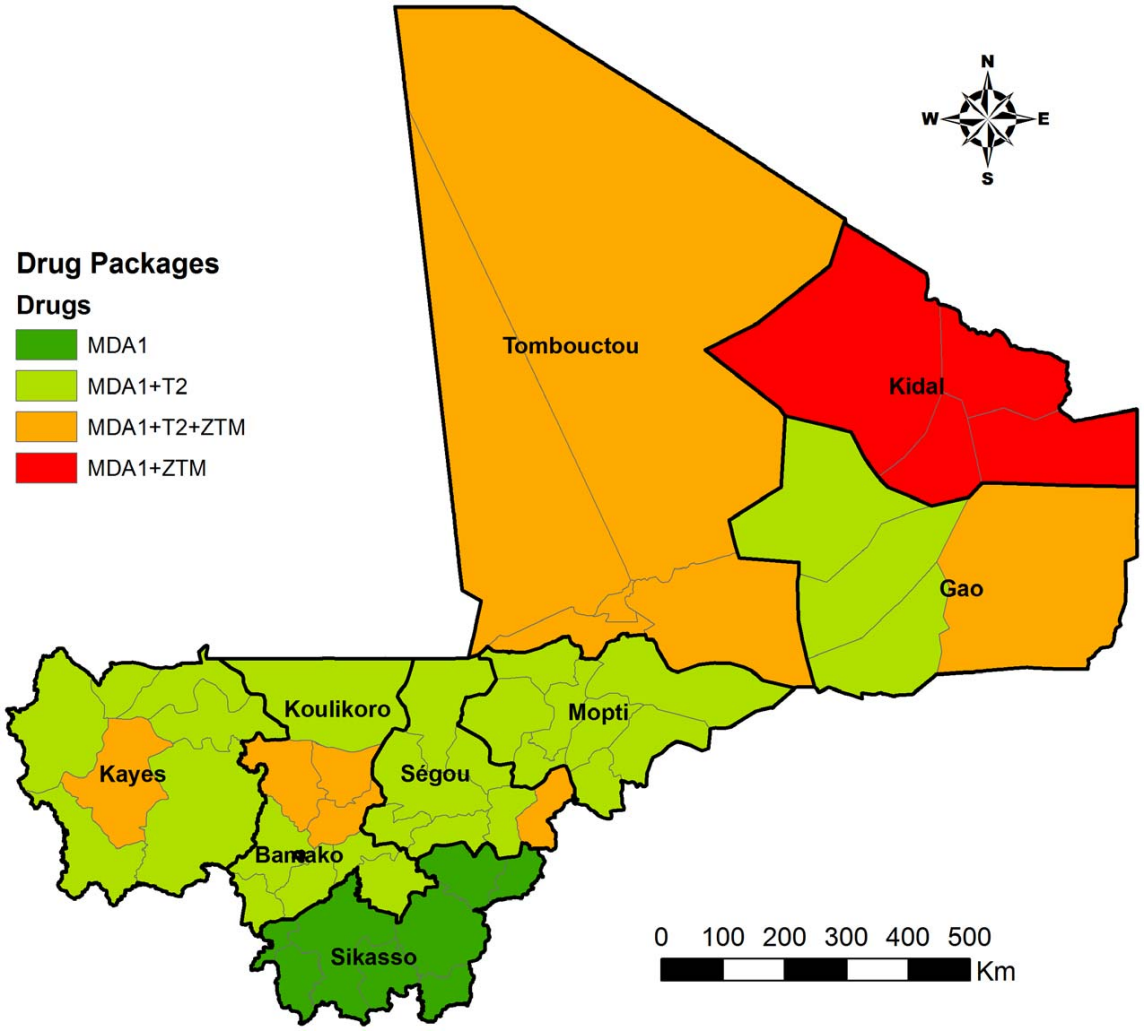
Drug packages required for each district according to the WHO PCT guidelines. MDA1: ivermectin+albendazole; T2: praziquantel, ZTM: Zithromax. In Kidal region the endemicity level of schistosomiasis in each district is not yet clear and further mapping is planned.

Drug packages for each district were determined as shown in Figure 3 according to the disease distribution shown in Figure 2 above and the WHO PCT guidelines. There was insufficient evidence and hence lack of clear guidance for combinations of available drugs, therefore, to avoid possible side effects due to combination, different drug packages were delivered in sequential fashion with one week between deliveries, where two or more drug packages were required. For example, where all three packages were required, MDA was organized as ZTM for week 1, ALB/IVM for week 3 and PZQ for week 5. This was also to avoid confusion among the CDDs with managing different dose poles at the same time, considering the relatively low education level in Mali villagers.

Several successful strategies for drug delivery were used by existing disease-specific national control programs, e.g. CDTI for onchocerciasis and LF, school-based and community-based drug delivery for schistosomiasis and STH, and community-based drug delivery for trachoma. Each of these was operating in disease-specific program areas. To scale up each program to a national coverage in the integrated control program, the four existing national programs worked together to plan and coordinate the MDA activities. A number of drug delivery strategies were used in combination in districts to deliver the drug packages by the trained CDDs: 1) School-based distribution by trained school teachers, taking place in schools targeting school-going children; 2) Community-based distribution by CDDs, including door-to-door/household distribution, focal distribution in the market, mosque, or other busy places, and mobile distribution through CDDs travelling on motorbike to households in remote areas, particularly in nomadic zones; and 3) Health center distribution by CHCWs, taking place at the health centers. Before MDA, in villages the trained CDDs work together with village chiefs to register the target population including name, age and sex. They receive drug allocations from community health centers according to the estimated population in each village and take the drugs to the village. CDDs, village chiefs and CHCWs discuss to decide the best drug delivery strategies for each village, mainly using community-based door-to-door distribution and if MDA happens during school terms, school-based distribution as well. In cities/towns, all the above mentioned three strategies are normally used. During MDA, CDDs distribute drugs according to the registered list, and they first confirm that the person has not been treated before treating him/her. The drugs administered are recorded in the register. MDA normally takes 2–3 days in each village. Extensive advocacy was conducted before each round of MDA and sufficient information was given to the general public about the national program. The drugs were voluntarily taken by the persons targeted in the endemic districts.

### Training and drug distribution

CDDs in each village were selected by the village and the management team of the community health center, and were used in the program to conduct the MDA activities in communities. The criteria for CDDs include: they were respected by the community; they had ability to read and write; and they were available during the MDA campaigns.

Cascade training for integrated drug administration was carried out throughout the implementation areas. The training sessions started at the regional level and cascaded down to the community level. Training of trainers was organized in the regions and these trainers subsequently trained the CHCWs (as supervisors) at the district level. The supervisors then trained the CDDs at the community health centers. Refresher training was also provided for supervisors and CDDs each year before the MDA campaign started. Table 1 shows the number of people trained or retrained from 2007 to 2011. In view of the usually low educational level of Mali villagers, the NTDCP decided to train the CDDs in the drug administration before each treatment round with different drug packages in order to avoid confusion in CDDs to calculate and administer the drugs using different dose poles. As the national program has matured and in efforts to reduce costs and streamline the program, integrated training is now being introduced. In total, 86,248 persons have so far been trained and retrained across the country.

**Table 1:** Number of persons trained under the integrated NTD control program (10.2007–03.2011).

Categories of trainees	Number of persons trained
Ministry of Health staff at central level	10
Trainers	373
Supervisors (CHCWs)	3,386
CDDs	81,194
Other ( staff from Academies of Teaching, Pedagogic Center of Animation, Social Development Services, NGOs in health sector, interviewers for surveys)	1,285
TOTAL	86,248

### Advocacy and community mobilization

Advocacy activities undertaken aimed to promote country ownership of the control program through increasing government funding and support to the NTDCP activities and to mobilize resources from existing and potential partners. At sub-country level, advocacy activities were focused on mobilizing support from local authorities at the regional, district and community levels.

Before each campaign, an official notice was sent by the National Director of Health to all Regional Directors of Health to inform them the mass treatment campaign and to request them to achieve the objectives of the program. Several meetings between the various stakeholders (Regional Directorates of Health, the Regional Offices of Education, Social Development, local councils) involved in the control of NTDs were conducted to galvanize interest, support and participation in the campaign. Posters were produced and sent to all health districts and radio and television messages were broadcast to announce the mass treatment campaign.

Meetings with local officials were held to mobilize communities during mass treatment campaign. Images of severe cases of each of the five NTDs were shown, including the short-and long-term signs and symptoms and the treatment available. These meetings also served as means of motivating communities to participate in the mass treatment campaign. These meetings also proved to be effective in mobilizing funds to support CDDs in some districts.

### Behavior change communications

Behavior change communication has been a very important part in the Mali integrated NTD control program. A workshop to develop and harmonize health messages was organized each year. It was followed by the development of audiovisual materials. These messages were broadcast on the various channels during the month immediately preceding the campaigns, and throughout the duration of the campaigns. Posters and banners were also posted strategically during the course of the weeks preceding the campaign. Short documentaries on NTDs and mass treatment campaigns were broadcast on television at least three times during the 20 days preceding the campaign as well as during the campaign. The same schedule was used for broadcasting the radio messages.

Counseling cards on the five NTDs were designed and these cards are used by the CDDs during mobilization and drug distribution to educate people about the disease and the importance of treatment. The cards also contained information for communities to understand the behaviors that could cause or complicate these diseases and the behaviors that could help prevent them from getting the diseases, such as hand washing and face washing. To date, 3,000 counseling cards and 500 posters have been produced.

### Data collection and analysis

Data on treatment and serious adverse events (SAEs) in this paper were the CDD-reported data from the NTDCP. During the mass treatment campaign, the CDDs recorded data on drug usage, treatment numbers and SAEs using specific reporting forms. The data were reported to national NTDCP through health reporting system. In 2009, the reported coverage data were verified through a post-PCT coverage survey (details not shown here). In the current paper, to standardize the calculation for all targeted NTDs, national census population was used and population at risk for each NTD was estimated according to the annual projected population figures from the National Directorate of Population, Mali. Eligible population was estimated as the total population at risk for trachoma and 80% of the total population at risk for LF, onchocerciasis, schistosomiasis and STH. The coverage rates were calculated according to the WHO guidelines for drug coverage monitoring, including geographical coverage, program coverage and national coverage [23]. The geographical coverage is the percentage proportion of the targeted districts among the total number of endemic districts for each disease. The program coverage is the percentage proportion of the population treated among the eligible population in the targeted program areas. The national coverage is the percentage proportion of the population treated among the total population at risk in the country.

The cost data were from the HKI program accounts specific for direct expenditure in Mali on the NTD program activities. HKI receives expense receipts after completion of each activity from the NTDCP. The original receipts for all expenses are maintained by HKI, and are spot checked during internal financial reviews as well as during HKI's federally-mandated annual A-133 audit. Expenses, such as vehicle fuel, per diems, and supplies etc. directly incurred during the implementation of program activities, are uniquely coded in HKI's financial system based on the type of activity supported (e.g. training of CDDs, drug transport and distribution, etc.). On a monthly basis, all program expenses are categorized by activities based on these unique codes, and a running cost total is maintained for each activity over the life of the project.

## Results

### Scale-up of geographical coverage

The integrated MDA activities started in 2007. To gain experience of the integrated delivery of different drug packages by the CDDs, the integrated drug delivery started in three regions (Kayes, Koulikoro and Sikasso) which included 24 districts. It was then gradually scaled up to include more regions in the following years to achieve national coverage in 2009. The number of districts covered by MDA each year since 2005 and the cumulative coverage are summarized in Table 2. Onchocerciasis MDA achieved 100% geographical coverage before 2007 and this has been maintained since. Trachoma MDA started in two regions (Kayes and Koulikoro) and already met the program target after three rounds of treatment before 2007. It was gradually expanded to include all other endemic regions in 2009. The significant gain of the integrated NTD program was the scale-up for LF MDA which achieved full national geographic coverage in 2009, and this has since been maintained. The national coverage of LF MDA also benefited STH control throughout the country. MDA for schistosomiasis achieved national coverage for school-age children in 2007, and each endemic district had been targeted two to five times by the end of 2010 according to the endemicity level. In the scarcely populated Kidal region, the mapping of schistosomiasis in this region was not conducted due to the insecurity and will be done later. MDA for schistosomiasis targeting school-age children in this region was delivered based on the historical and clinical knowledge.

**Table 2:** Number of districts targeted annually for MDA for each disease since 2005.

Diseases	2005	2006	2007	2008	2009	2010	2011^a^
LF	4(6.8)^c^	15(25.4)	24(40.7)	35(59.3)	59(100)	59(100)	60^b^(100)
Onchocerciasis	16(100)	16(100)	16(100)	16(100)	16(100)	16(100)	17^b^(100)
Schistosomiasis	31(58.5)	40(75.5)	28(100)	45(100)	27(100)	49(100)	23(100)
STH	31^d^(52.5)	40^d^(67.8)	24(78)	35(86.4)	59(100)	59(100)	60^b^(100)
Trachoma	19(38)	24(64)	8(80)	29(90)	38(100)	23(100)	10(100)

a 2011 MDA was still ongoing during drafting of this paper so these are projected figures.

b Sikasso district in the original 59 was split into two districts to become a total of 60 districts.

c Figures in brackets represent the cumulative geographical coverage.

d Figures include the districts where schoolchildren were treated with ALB together with schistosomiasis MDA.

### Scale up of program coverage

With gradual scale-up of geographical coverage, the number of people targeted and treated/retreated each year increased noticeably. The annual treatment numbers for each targeted NTD and the percentage coverage (program coverage and national epidemiological coverage) rates are shown in Table 3 (including data from 2005–06 before integration). Overall, satisfactory program coverage rates had been achieved each year in the targeted areas since 2007 and maintained at high level since 2009, with those for LF, onchocerciasis, STH and trachoma ranging from 76% to over 100%. Although overall program coverage rate for schistosomiasis was relatively lower each year, program coverage rates had been high among school-age children, the main targeted group according to the WHO recommendations. Most notably, the national epidemiological coverage for LF steadily increased over the years to reach over 65%, treating around 10 million people each year since 2009, and this also benefited STH control throughout the country with national epidemiological coverage of 66–75%.

**Table 3:** Annual number of persons treated and treatment coverage for the targeted NTDs.

	2005	2006	2007	2008	2009	2010^a^
**LF**						
No of persons treated	511,416	2,216,705	4,527,977	5,445,651	9,762,073	10,047,125
Program coverage (%)	103.9	97.6	94.9	84.7	91.5	91.1
National coverage (%)	4.4	18.5	36.8	43.1	75.3	66.8
**Onchocerciasis**						
No of persons treated	1,587,000	1,734,586	1,635,416	1,701,300	3,069,804	3,387,412
Program coverage (%)	101.8	101.6	94.5	78.7	103.8	90.0
National coverage (%)	39.6	42.2	38.7	39.3	69.0	65.9
**Trachoma**						
No of persons treated	3,334,845	5,383,845	1,767,877	5,537,302	6,416,637	3,145,826
Program coverage (%)	79.8	104.9	78.6	76.2	78.5	97.2
National coverage (%)	32.4	51.2	16.5	50.4	57.2	23.9
**Schistosomiasis**						
No of persons treated	2,598,138	2,174,940	895,343	2,551,995	1,796,586	4,526,684
(No of SAC^b^ treated)	(1,948,964)	(1,627,240)	(384,104)	(1,468,410)	(1,148,733)	(2,704,918)
Program coverage (%)	50.0	40.1	47.8	58.2	71.4	69.5
(Program coverage SAC (%))	(99.0)	(80.8)	(72.7)	(100.7)	(92.9)	(88.8)
National coverage (%)	22.2	18.1	7.3	20.2	13.9	30.1
(National coverage SAC (%))	(55.6)	(45.2)	(10.4)	(38.8)	(29.5)	(60.0)
**STH**						
No of persons treated	2,598,138	2,174,940	4,427,977	5,445,651	9,762,073	10,047,125
Program coverage (%)	48.8	40.5	94.9	84.7	91.5	91.1
National coverage (%)	22.2	18.1	36.8	43.1	75.3	66.8

a Population in 2010 was projected according to the 2009 census while populations for previous years were projected according to the 2000 census.

b SAC – school-age children.

### Severe adverse effects

Some minor side effects from taking the drugs had been recorded such as diarrhea and headaches and these were usually dealt with at the community health centers. However, no cases with severe adverse effects have been recorded so far.

### Cost of the program

**Figure 4:**
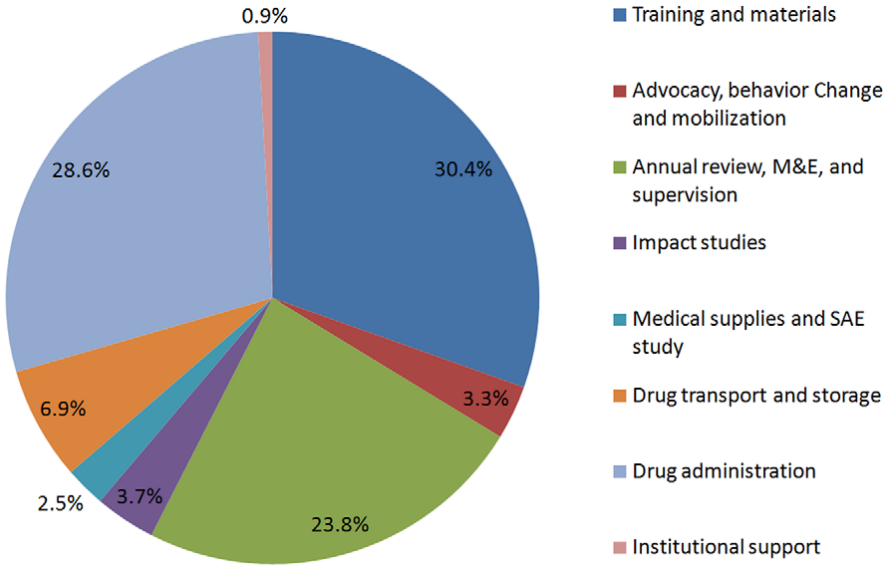
Percentage expenditure of the NTD program activities for the first four rounds of MDA.

The total direct cost of the program in Mali is $3.575 million from the start of the program in 2007 to March 2011, which covers four rounds of drug delivery. The cost shown here does not include the significant contribution from the MoH on housing, logistics, staff salaries etc., and the cost of drugs, which were either donated free of charge or directly procured by RTI. It also does not include any opportunity costs and monetary contributions from local governments, for example, in 2010 the Kayes mayor's office donated an amount of five million francs (CFA) to help motivate the CDDs during the campaign. As expected, the major expenditures were for MDA activities which included training of CDDs, drug transport, storage and administration, and M&E, supervision and annual program reviews (Figure 4).

## Discussion

The concept of integration was to increase efficiency and treatment coverage to deliver the drugs to those who are in need. Since the injection of major funds from the USAID for the integrated NTD control activities, Mali has significantly scaled up MDA coverage for all five targeted major NTDs. Apart from the success in onchocerciasis control already achieved, national geographical coverage has been achieved for LF from 25% in 2006 to 100% in 2009, reaching national coverage of over 65%. After completion of four rounds of MDA, a sentinel site study was conducted in Sikasso and Koulikoro regions in 2010, and will be conducted in other regions accordingly when the criteria are met in each region. The results will be published separately once enough data are collected. Such national coverage of treatment with ALB and IVM for LF greatly benefits the STH control throughout the country. It also continues to benefit the onchocerciasis elimination in the original 16 districts at risk for onchocerciasis. A recent publication from multi-center studies including the Bakoye river focus in Mali showed that the prevalence of onchocerciasis had been reduced to below 1% by 14 years of annual treatment, which confirmed the feasibility of elimination of the disease in Africa [24]. Impact studies in other areas are to be conducted, and Mali is on track to achieve the objective of elimination of onchocerciasis as public health problem which has recently been adopted by the African Program for Onchocerciasis Control as the program objective of onchocerciasis control in Africa [25].

Trachoma control is one of the most successful control programs in the country. Since integration, program coverage rates for trachoma have been very high, ranging from 76% to 97%. The PNLC through the NTDCP has made great progress with determining which districts no longer warrant treatment at district level by conducting impact studies after three to four consecutive rounds of treatment with Zithromax and tetracycline ointment. Currently only 10 districts in the five regions of Gao, Kidal, Koulikoro, Kayes, and Segou still warrant for MDA at district level. The PNLC is working with WHO, HKI, and the Carter Center to pilot a post-endemic surveillance protocol in the districts where trachomatous follicular (TF) prevalence has fallen below 5%. The relatively low annual national coverage for trachoma shown here was mainly due to the progressive starting of the MDA among districts and stopping of MDA in districts that no longer required treatment.

Treatment with PZQ for schistosomiasis at a national level has reached all eight regions plus Bamako with overall program coverage rates ranging from 40% to around 70%. The target population for schistosomiasis treatment in Mali has been primarily school-age children and adults at high risk. Program coverage in school-age children has been maintained over 80% except 72.7% in 2007. According to the WHO guidelines [4], not every district and child require annual treatment, therefore the annual national coverage rates shown here looked relatively low, even though the program coverage for school-age children each year was high. In addition to the USAID funding, Mali also receives support from other partners in country, such as the Organization for the Maintenance of the River Senegal (OMVS) and World Vision International (WVI), whose funds helped to procure PZQ and support distribution costs in the regions of Kayes and Koulikoro.

Integration of vertical national control programs was complex and challenging [26]. As one of the first five ‘fast-track’ countries to implement the integrated program without prior examples to follow, Mali designed and adopted the implementation strategies according to the local context. The program was coordinated by the national NTDCP team and at the local level it was integrated into the primary health care system. The NTD control became a routine activity of the CHCWs. There was a concern that involvement of the CHCWs would impact on their time in responding to curative cares hence interrupting the service provision at the local health facilities [27]. This may have been the case at the beginning of the program; however, this approach would provide the best chance for long-term sustainability of NTD controls [3], [28]. Over 3000 CHCWs countrywide have been trained and retrained, who will be able to provide quality health services at health centers for NTDs once the large-scale MDA is scaled down when the short-term objectives are met. In reality, it is anticipated that such a large-scale intervention would have reduced the demand of the CHCWs' time due to the reduction of morbidity reverted by MDA [29], [30].

Despite the progress of the NTD control program and achievements made, there are a number of difficulties/challenges still in the program and lessons learnt:

1. For the long-term sustainability, the program is integrated with the primary health care system at the local level. At this level there are many overlapping public health campaigns that occur at the same time as the MDA, such as national immunization days. Competition for the CHCW and CDD time is high, which means possible delay for one program or another. Better coordination is needed, perhaps further integration of MDA. A study has been carried out to use the SIAN program as a possible platform for MDA. A further combination of drugs may also reduce the need of CHCW and CDD time by reducing the number of drug packages and rounds of drug delivery. A number of studies showed that combining ALB, IVM and PZQ caused no more severe adverse effect than the current drug combinations [31], [32], and such further combination will be considered in the future MDAs as experience has been gained by the CDDs. Study on combining Zithromax with ALB and IVM is also underway in Mali, which will pave the way for further combination in non-schistosomiasis MDA areas.
2. Like in many other countries [33], motivation of CDDs has increasingly become a challenge. It is difficult to retain CDDs without financial incentives for NTD programs while other programs e.g. those for malaria and HIV/AIDS are paying them. Traditional kinship structure has successfully been used to enhance the MDA for onchocerciasis in Uganda [34]. As mentioned earlier, the Kayes mayor's office set an example in donating funds to help motivate the CDDs during the campaign, and such local contributions may be sought in future in other regions.
3. With the current drug donation mechanisms involving multiple donors and multiple levels of drug management, delay in drugs reaching the country for distribution has been a challenge and may remain so in near future. Such delay often causes MDA postponed into rainy season, which not only increases the difficulty in the campaign but also minimize the impact of MDA, particularly for schistosomiasis. Forward planning and drug procurement at the country program level is necessary to overcome such obstacles. The NTDCP has therefore now decided to revise its annual program so that all the key events are scheduled to take place earlier than previously or usually planned. The annual review, which usually takes place in November, will now take place at the end of September. This will enable to the program to get the final MDA results earlier and, as a result, to place drug orders earlier.
4. For a long-term, sustainable control/elimination of NTDs, comprehensive measures are needed. The current NTD funds are almost exclusively supporting the integrated MDA. Case management, clean water supply, hygiene and sanitation, and vector management etc. are all critical components of a control program in order to achieve and maintain the long-term program objectives. The NTDCP received funds from Conrad N Hilton Foundation through HKI and the Carter Center to implement the S, F and E components of the trachoma SAFE strategy, but so far are struggling to find funds to support the non-MDA components for other NTDs.
5. Funds are available for the monitoring and evaluation on the impact achieved for LF and trachoma, but there are insufficient funds to support similar activities for other three targeted NTDs. As described, Mali has conducted several rounds of integrated MDA for onchocerciasis, schistosomiasis and STH. There is a need to reassess the prevalence level throughout the country in order to adjust the MDA strategy.
6. It has been a challenge to collect data from all districts after each MDA in a timely fashion. Nationally the integrated MDA is a long campaign each year lasting around five months from training to national annual review. Any delay in collecting data from each district will affect the annual planning for the next year. A successful pilot trial using mobile phone text messages to report the MDA data was conducted in a number of districts. This may be expanded in the country to expedite the data reporting in future.
7. Inventory keeping and re-stocking of residual drugs after MDA has been difficult. This resulted in significant loss of residual drugs. The supply chain management needs to be strengthened. Assistance from external consultants has been sought, and it is hoped that the system should be improved in next rounds of MDA.
8. Mali is a large country deep into the Sahara desert. It has been a real challenge for the integrated MDA to reach remote areas, particularly, nomadic populations in the northern half of the country (e.g. Gao, Kidal, and Tombouctou). These areas are also insecure which often cause delay in MDA. To overcome these, the NTDCP used various drug delivery strategies within the regions, particularly e.g. the mobile teams (CDDs on motorbikes) to reach those in need.
9. At the beginning of the program, local people had little knowledge about NTDs and complications, and therefore the compliance of MDA was low. The NTDCP used the visual aids (posters, counseling cards etc.) containing pictures of severe NTD cases, in particular LF and schistosomiasis, to educate and mobilize the populations. The program also involved the political leaders, religious leaders and village chiefs in starting the MDA campaign. Their taking of the drugs set a great example for community members to follow.

### Conclusions

Built on the existing success of individual national control programs, the Government of Mali has shown commitment in the control of NTDs in the country. The coordination of NTD control has been integrated at the central level and implementation activities are integrated with the primary health care system at the local communities. With the financial support from the USAID and other donors, Mali has scaled up the drug treatment to a national coverage through integrated drug delivery, with around 10 million people receiving one or more drug packages each year since 2009. With the progress of the program, the focus is now on consolidating the achievements to achieve the goals of eliminating LF and blinding trachoma, perhaps also onchocerciasis, and reducing the morbidity caused by schistosomiasis and STH, in the country by the preset timelines, and on mobilizing resources for the next phase of the NTD control according to the new national strategic plan. Mali's successes and lessons learned can be valuable assets to other countries starting up their integrated national NTD control programs.
